# The significance of homeodomain transcription factor 2 in colon cancer cells

**DOI:** 10.1186/s12938-021-00912-5

**Published:** 2021-08-09

**Authors:** Yang He, Peng Gong, Sitong Wang, Qing Xu, Jianhua Chen

**Affiliations:** 1 Department of Oncology, Shanghai Tenth People’s Hospital, Tongji University School of Medicine, 301 Yanchang Road Middle, Shanghai, 200072 People’s Republic of China; 2 Department of Interventional Oncology, Shanghai DaHua Hospital, Shanghai, 200072 People’s Republic of China

**Keywords:** Colon cancer, Paired-like homeodomain transcription factor 2, Long non-coding RNA gastric carcinoma high expressed transcript 1, Wnt/β-catenin pathway, Proliferation, Invasion

## Abstract

**Background:**

Colon cancer is a serious malignant tumor. It has been reported that paired-like homeodomain transcription factor 2 (PITX2) can promote the progression of several types of cancer via regulating the Wnt/β-catenin pathway. It has also been demonstrated that high levels of long non-coding RNA (lncRNA) gastric carcinoma high expressed transcript 1 (GHET1) can also promote the development of cervical cancer via activating the Wnt/β-catenin pathway. However, whether PITX2 can affect the development of colon cancer via regulating the expression of lncRNA GHET1 remains unclear.

**Results:**

The results demonstrated that PITX2 knockdown attenuated the proliferation, migration and invasion abilities of colon cancer cells. Additionally, PITX2 promoted the expression of lncRNA GHET1 via binding to its promoter. Overexpression of lncRNA GHET1 induced the expression of Wnt/β-catenin signaling-related proteins, cyclin D1, c-Myc and MMP-7. Furthermore, lncRNA GHET1 overexpression abrogated the PITX2 silencing-mediated decreased proliferation, migration and invasion abilities of colon cancer cells.

**Conclusion:**

The findings of the present study suggested that PITX2 could enhance the proliferation, migration and invasion abilities of colon cancer cells via upregulating lncRNA GHET1 and activating the Wnt/β-catenin pathway.

## Background

Colon cancer is one of the most common malignant tumors worldwide and its mortality and morbidity rates have been increasing in recent years [1]. The occurrence and development of colon cancer has also become a major public health concern worldwide [2]. Unhealthy lifestyle habits are considered as major contributors to the occurrence and development of colon cancer [3]. Furthermore, the combination of surgery with chemotherapy is considered as the most commonly used strategy for patients with colon cancer [4]. In recent years, the continuous development of technology has provided improved therapies for colon cancer. However, the therapeutic outcome of patients with colon cancer remains unsatisfactory. Therefore, further studies are urgently needed to explore the molecular mechanisms underlying the development of colon cancer to provide novel targets and strategies for the clinical treatment of this disease.

Paired-like homeodomain transcription factor 2 (PITX2) is a member of the bicoid-like homeobox family and plays a crucial role in vertebrate embryogenesis [5]. It has been reported that PITX2 may be associated with the onset of multiple sclerosis [6]. In addition, other studies demonstrated that PITX2 was significantly upregulated in thyroid and colorectal cancer tissues, thus promoting the development of these malignant tumors [7, 8]. PITX2 could activate the Wnt/β-catenin signaling pathway via upregulating Wnt family member 3A (WNT3A), thereby promoting the development of lung adenocarcinoma [9]. Another study revealed that, during the development of colon cancer, the expression of PITX2 may enhance the proliferation and metastasis of colon cancer cells, while it was found to be associated with decreased survival rates among patients with colon cancer [10].

Long non-coding RNAs (lncRNAs) are a class of RNA molecules > 200 nucleotides in length [11]. Emerging evidence has suggested that lncRNAs are involved in the regulation of proliferation and metastasis of various tumor cells [12, 13]. The lncRNA gastric carcinoma high expressed transcript 1 (GHET1) has been found to be involved in the development of various types of cancer [14, 15]. A previous study demonstrated that knockdown of lncRNA GHET1 inhibited the proliferation and metastasis of non-small lung cancer cells [16]. In addition, lncRNA GHET1 silencing attenuated the proliferation and invasion of colorectal cancer cells [17]. Interestingly, the expression of lncRNA GHET1 could also promote the progression of cervical cancer via activating the Wnt/β-catenin pathway [18]. Therefore, the current study hypothesized that PITX2 could activate the Wnt/β-catenin pathway via regulating the expression of lncRNA GHET1 to promote the occurrence and development of colon cancer. Herein, the effect of PITX2 and lncRNA GHET1 on the proliferation, migration and invasion of colon cancer cells was evaluated, in the hope of providing novel targets and strategies for colon cancer.

## Results

### PITX2 knockdown suppresses colon cancer cell proliferation

**Fig. 1:**
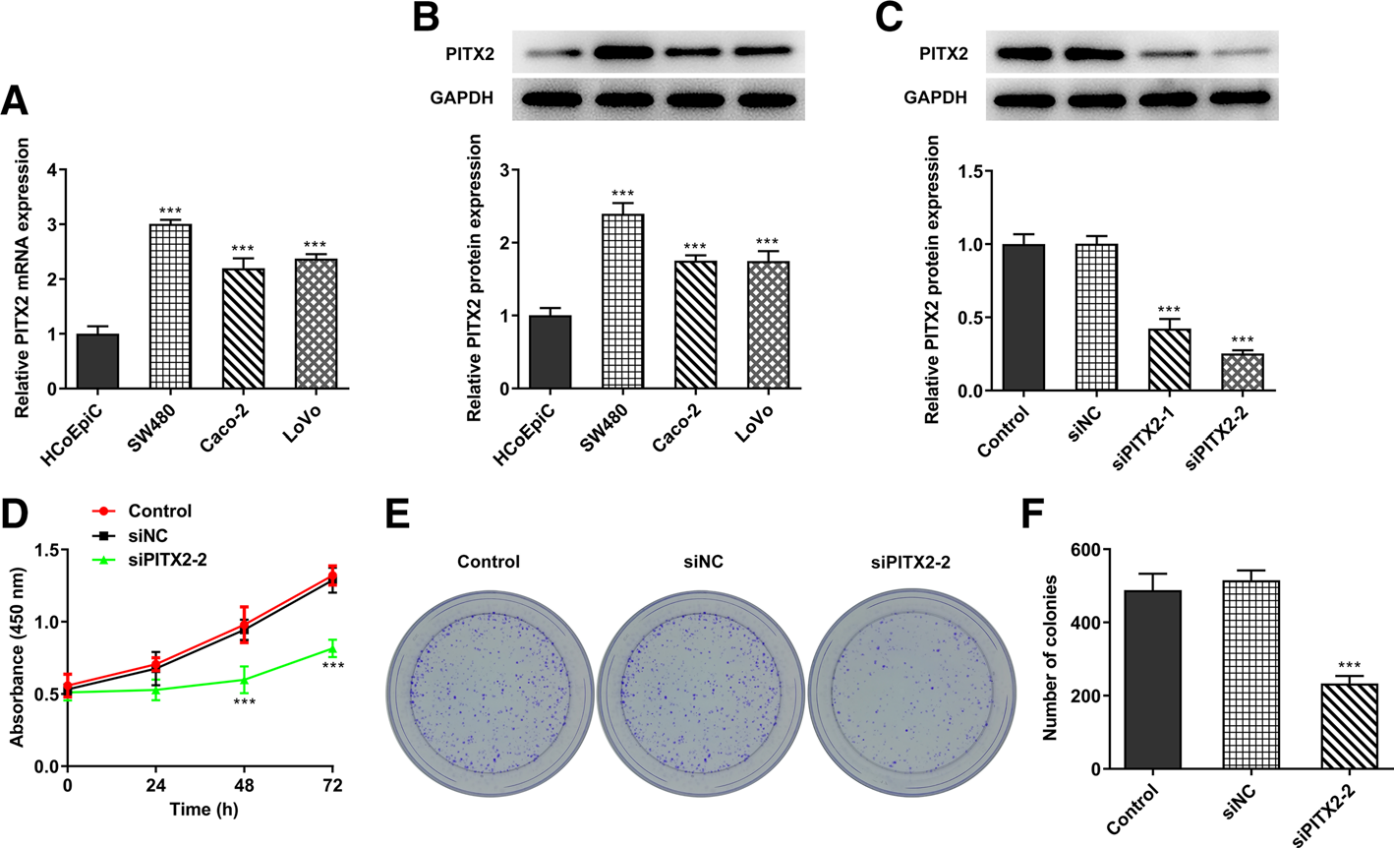
Knockdown of lncRNA PITX2 suppressed the proliferation of colon cancer cells. **A**, **B** The expression of PITX2 in colon cancer cells was detected with the RT-PCR and western blotting. **C** The expression of PITX2 in SW480 cells was determined with the western blotting. **D** CCK-8 assays were performed to detect the proliferation of SW480 cells. **E**, **F** Colony formation assay was performed to explore the proliferation of SW480 cells. ***p < 0.001 vs. HCoEpiC or control

To evaluate the effect of PITX2 on the development of colon cancer, the expression levels of PITX2 were determined in HCoEpiC colonic epithelial cells and the colon cancer cell lines, SW480, Caco-2 and LoVo, by RT-qPCR and western blot analysis. The results showed that PITX2 was upregulated in colon cancer cells compared with the normal colonic epithelial cells (Fig. 1A, B). Since SW480 exhibited the highest PITX2 expression, these cells were selected to establish PITX2-knockdown colon cancer cells. As shown in Fig. 1C, PITX2 was downregulated in the knockdown group. Additionally, the inhibitory effect of small interfering (si)PITX2-2 on PITX2 expression was more potent compared with that of siPITX2-1. Therefore, the siPITX2-2 clone was selected for subsequent experiments. Furthermore, CCK-8 and colony formation assays were performed to evaluate the effect of PITX2 silencing on SW480 cell proliferation. The results demonstrated that the cell viability and number of formed colonies were decreased following PITX2 knockdown (Fig. 1D–F).

### PITX2 knockdown attenuates the migration and invasion of colon cancer cells

**Fig. 2:**
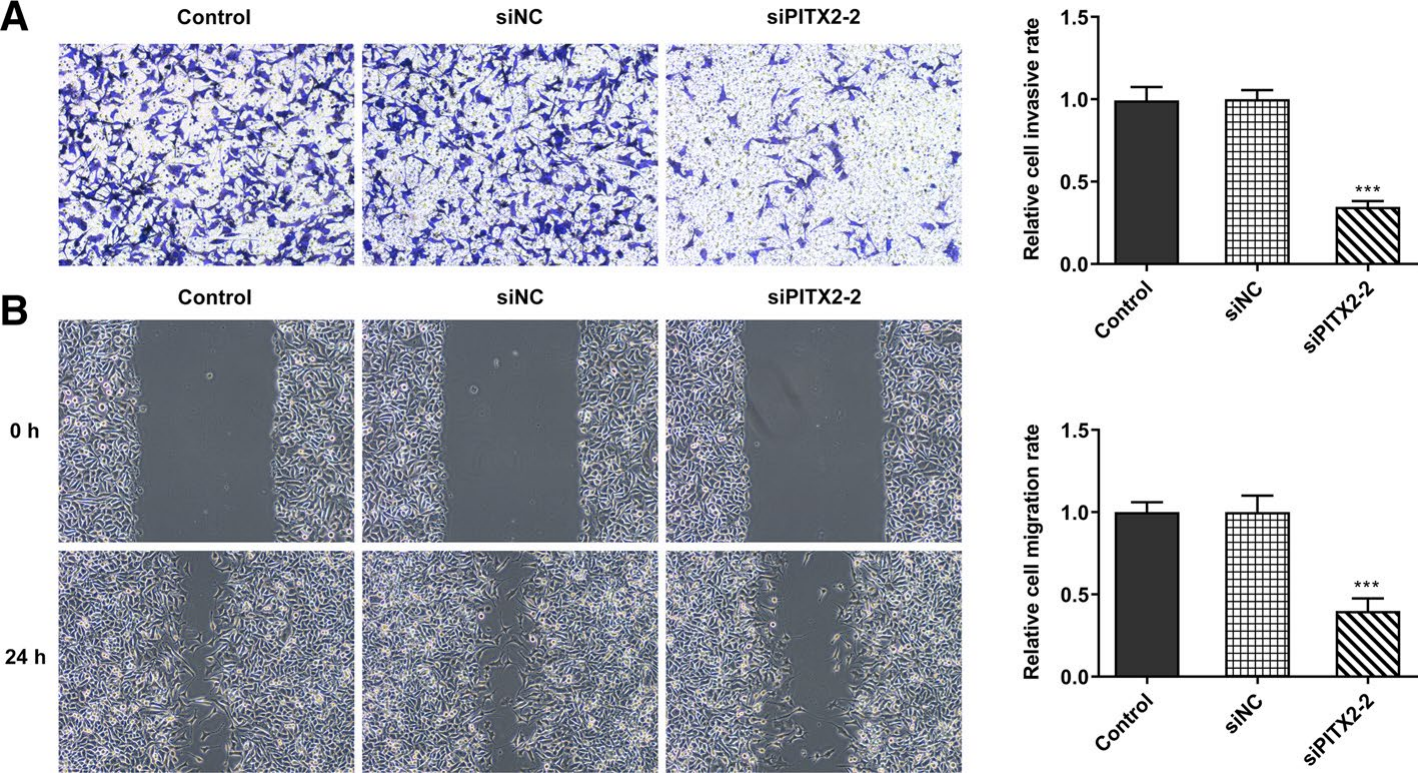
Knockdown of lncRNA PITX2 inhibited the migration and invasion of colon cancer cells. **A** Transwell was performed to detect the invasion of SW480 cells. **B** Wound healing assay was performed to detect the migration of SW480 cells. ***p < 0.001 vs. control

Subsequently, the effect of PITX2 silencing on colon cell migration and invasion was evaluated. The Transwell assay revealed that the number of invaded colon cancer cells was decreased after PITX2 knockdown (Fig. 2A). Similarly, wound healing assays showed that the migration ability of colon cancer cells was reduced following PITX2 knockdown (Fig. 2B).

### PITX2 promotes the expression of lncRNA GHET1

**Fig. 3:**
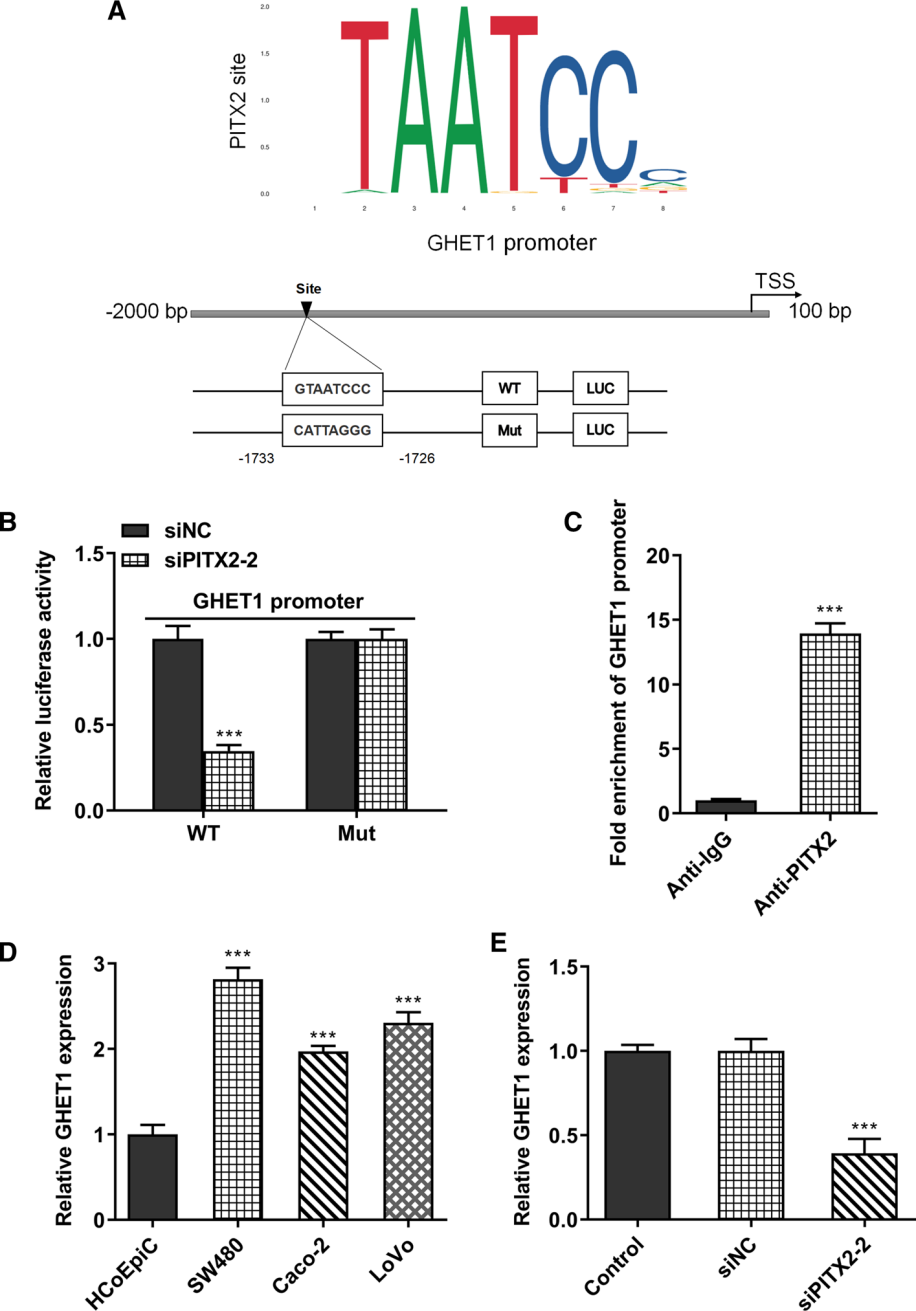
PITX2 bound the promoter region of lncRNA GHET1 and promoted the expression of lncRNA GHET1. **A** JASPAR analysis (http://jaspar.genereg.net/) predicted revealed that the expression of PITX2 was associated with that of lncRNA GHET1. **B** Luciferase reporter assay was performed to detect the relationship between PITX2 and lncRNA GHET1. **C** CHIP assay was performed to explore the binding between PITX2 and lncRNA GHET1. **D** The expression of lncRNA GHET1 in colon cancer cells was determined with the RT-PCR. **E** The expression of lncRNA GHET1 in SW480 cells was detected with the RT-PCR. ***p < 0.001 vs. HCoEpiC or control

JASPAR analysis (http://jaspar.genereg.net/) predicted revealed that the expression of PITX2 was associated with that of lncRNA GHET1 (Fig. 3A). Subsequently, the association between PITX2 and lncRNA GHET1 was verified by luciferase reporter assay. The results showed that the fluorescence intensity was reduced following PITX2 knockdown (Fig. 3B). Additionally, ChIP assays also revealed that PITX2 could bind with the promoter region of lncRNA GHET1 (Fig. 3C). Furthermore, the expression levels of lncRNA GHET1 were measured in colon cancer cells. lncRNA GHET1 was upregulated in colon cancer cells compared with colonic epithelial cells (Fig. 3D). Interestingly, the expression of lncRNA GHET1 was suppressed in SW480 cells following transfection with siPITX2 (Fig. 3E).

### lncRNA GHET1 overexpression promotes the activation of the Wnt/β-catenin pathway

**Fig. 4:**
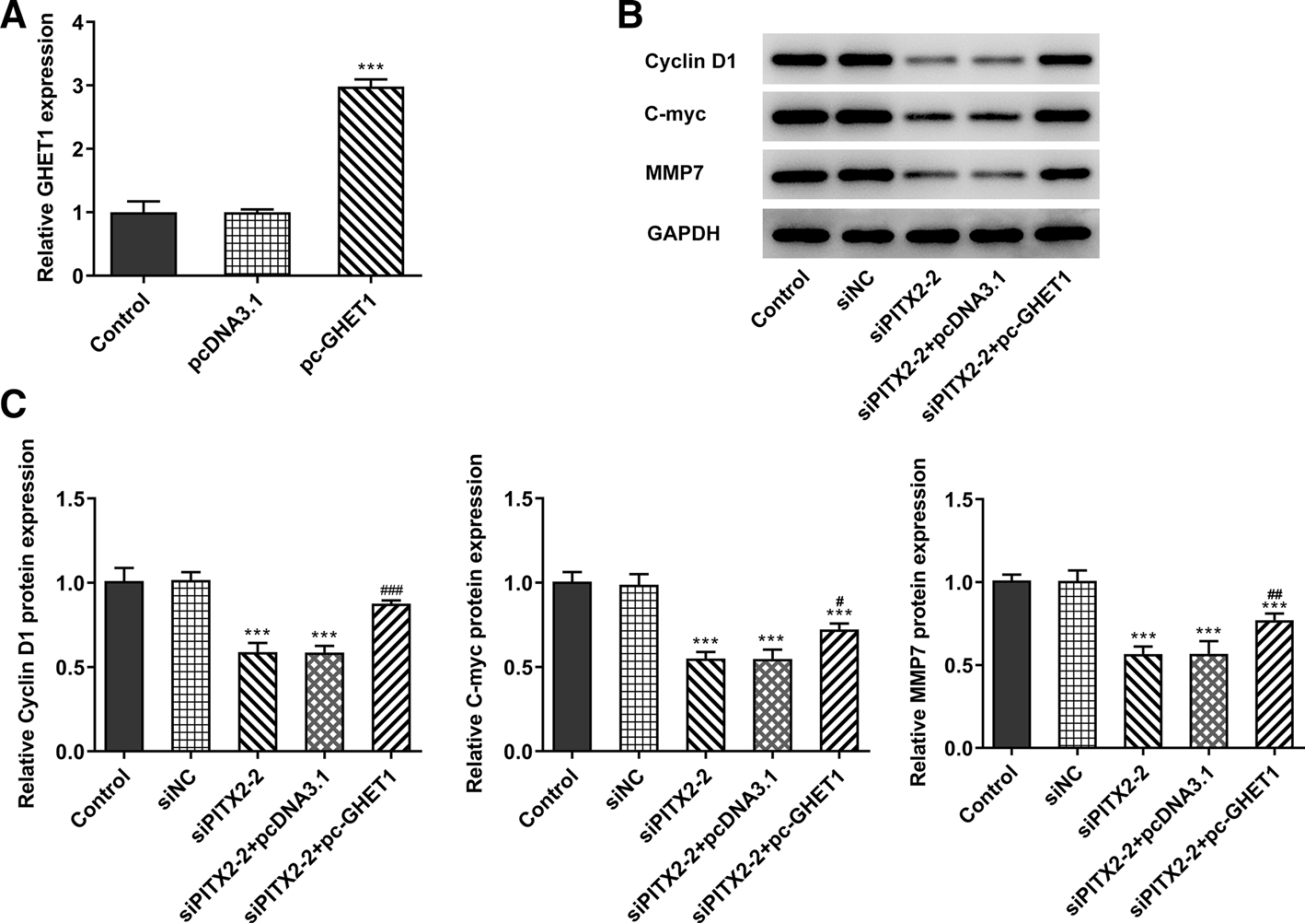
Overexpression of lncRNA GHET1 activated the expression of cyclin D1, c-Myc and MMP-7. **A** The expression of lncRNA GHET1 in SW480 cells was detected with the RT-PCR. **B**, **C** The expression of cyclin D1, c-myc and MMP-7 was determined with the western blotting. ***p < 0.001 vs. control; ^#^*p* < 0.05, ^##^*p* < 0.01, ^###^*p* < 0.001 vs. siPITX2-2

Subsequently, lncRNA GHET1-overexpressing SW480 cells were established and the RT-qPCR results confirmed that the expression of lncRNA GHET1 was increased in these cells (Fig. 4A). A previous study demonstrated that lncRNA GHET1 could affect the development of cervical cancer via modulating the Wnt/β-catenin signaling pathway [18]. Herein, the expression levels of the Wnt/β-catenin pathway-related proteins were determined. The results of western blotting demonstrated that the expression of cyclin D1, c-Myc and MMP-7 was inhibited following PITX2 silencing (Fig. 4B, C). However, this effect was reversed following lncRNA GHET1 overexpression.

### lncRNA GHET1 overexpression relieves the PITX2 knockdown-induced restriction of colon cancer cell proliferation, migration and invasion

**Fig. 5:**
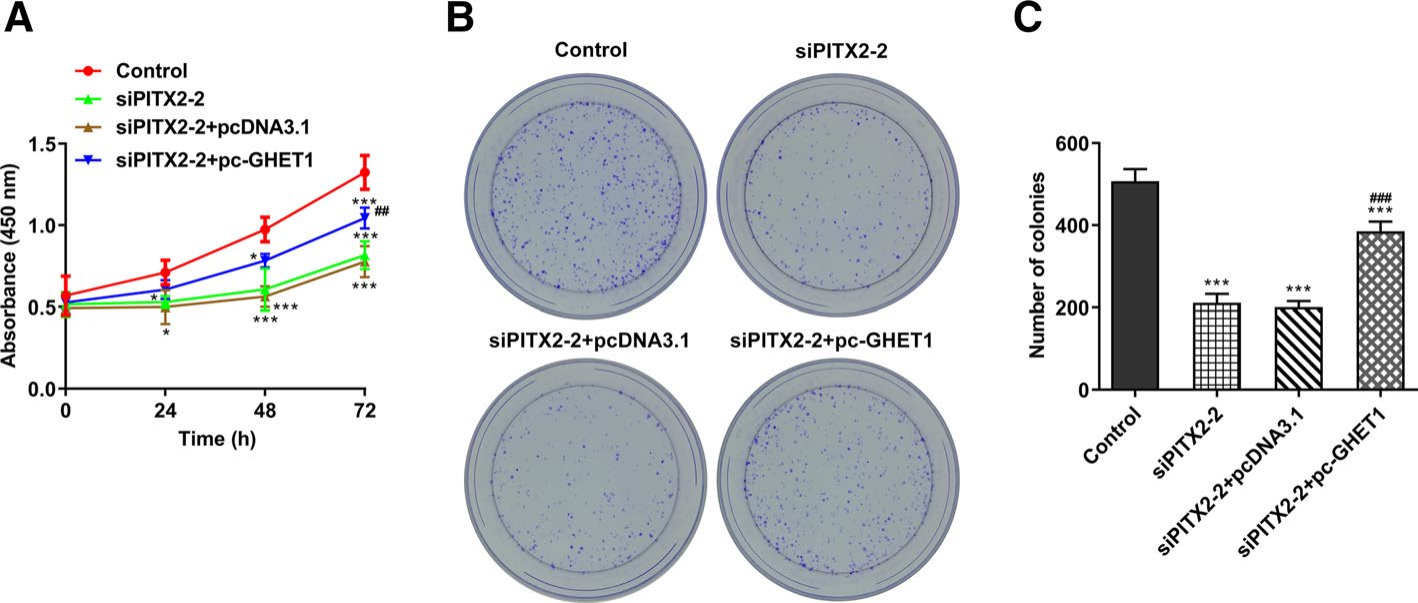
Overexpression of lncRNA GHET1 promoted the proliferation of colon cancer cells. **A** The proliferation of SW480 cells was detected with the CCK-8 assays. **B**, **C** The proliferation of SW480 cells was explored with the colony formation assay. *p < 0.05, ***p < 0.001 vs. control; ^##^*p* < 0.01, ^###^*p* < 0.001 vs. siPITX2-2

**Fig. 6:**
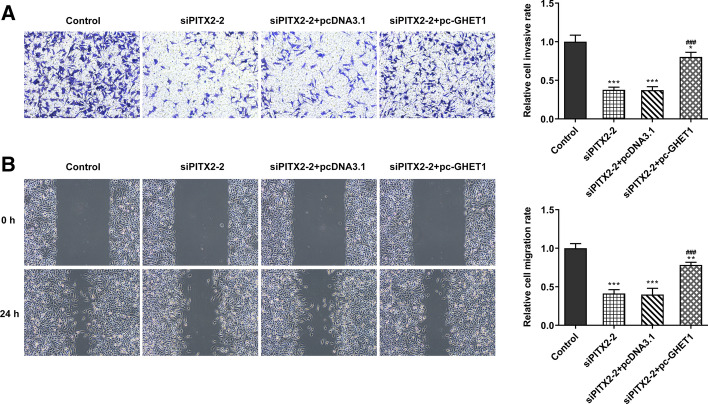
Overexpression of lncRNA GHET1 promoted the migration and invasion of colon cancer cells. **A** Transwell assay was performed to determine the invasion of SW480 cells. **B** Wound healing assays were performed to detect the migration of SW480 cells. *p < 0.05, **p < 0.01, ***p < 0.001 vs. control; ^###^*p* < 0.001 vs. siPITX2-2

The proliferative ability of SW480 cells was assessed by CCK-8 and colony formation assays. The SW480 cell viability and number of formed colonies were increased following lncRNA GHET1 overexpression (Fig. 5A–C). Similarly, Transwell and wound healing assays revealed that the overexpression of lncRNA GHET1 relieved the PITX2 silencing-mediated reduced migration and invasion abilities of colon cancer cells (Fig. 6A, B).

## Discussion

Colon cancer is one of the most common malignant tumors, and it is characterized by a high fatality rate and increasing incidence worldwide [19]. It has been reported that the dysregulated expression of several lncRNAs is associated with enhanced proliferation and metastasis of several types of malignant tumor cells [20]. Therefore, it is urgent to improve the treatment strategy for patients with colon cancer in order to increase their survival rate and quality of life. Therefore, identifying lncRNAs associated with the onset of colon cancer may provide novel targets for the treatment of patients with colon cancer.

It has been reported that PITX2 is involved in the development of several types of cancer [21, 22]. A study demonstrated that the PITX2 upregulation could enhance the resistance of breast cancer cells to letrozole via activating the interferon‑induced transmembrane protein 1 pathway [23]. Another study revealed that microRNA-21 could enhanced the apoptosis and attenuate the proliferation of pituitary adenoma cells [24]. Furthermore, PITX2 could also promote the development of lung adenocarcinoma via enhancing the expression of WNT3A and activating the Wnt/β-catenin pathway [9]. PITX2 upregulation was also reported to be associated with poor prognosis in patients with colorectal cancer [10]. In addition, PITX2 enhanced the ABC drug resistance of colon cancer cells via inversely regulating the human OCT3 (gene, Slc22a3) protein and ATP-binding cassette drug transporters [25]. Herein, PITX2 silencing also enhanced the reduced proliferation, migration and invasion abilities of colon cancer cells.

Emerging evidence has suggested the expression levels of several lncRNAs, a type of non-coding RNAs, can be used for the early diagnosis of different types of cancer [26, 27]. Therefore, lncRNAs have become a highly studied topic in the field of tumor research. It has been suggested that lncRNAs are involved in tumor progression via modulating the survival, proliferation and metastasis of tumor cells [28, 29]. Several studies have demonstrated that lncRNA GHET1 promotes cell proliferation, migration and invasion in different types of cancer [14, 30]. A previous study revealed that lncRNA GHET1 could promote the proliferation of ovarian cancer cells via enhancing glycolysis [31]. Additionally, lncRNA GHET1 could accelerate the development of cervical cancer via modulating the AKT/mTOR and Wnt/β-catenin signaling pathways [18]. Knockdown of lncRNA GHET1 could also suppress the proliferation and metastasis of colorectal cancer cells [17]. Since both PITX2 and lncRNA GHET1 could promote the proliferation of tumor cells via activating Wnt/β-catenin signaling, the current study hypothesized that both molecules could interact with each other. The results of the present study demonstrated that PITX2 could bind to the promoter region of lncRNA GHET1, thus promoting its expression. The activation of the Wnt/β-catenin pathway is characterized by the increased expression of cyclin D1, c-Myc and MMP-7 [32]. Herein, the expression of these proteins was found to be upregulated in colon cancer cells following lncRNA GHET1 overexpression. These findings also indicated that lncRNA GHET1 overexpression could activate the Wnt/β-catenin pathway and promote the proliferation, migration and invasion of colon cancer cells.

## Conclusions

The current study suggested that PITX2 could promote the proliferation, migration and invasion of colon cancer cells via enhancing the expression of lncRNA GHET1. Furthermore, lncRNA GHET1 overexpression could activate the Wnt/β-catenin pathway, thus promoting the proliferation and invasion of colon cancer cells. Overall, the results indicated that PITX2 may serve as a potential therapeutic target for colon cancer. Further study of the mechanism of action of PITX2 may have the possibility of clinical application, providing more options for clinical treatment. However, due to the limitation of time and funds, we have only based on SW480 cells to explore the PITX2 and lncRNA GHET1 regulated colon cancer progression. Further researches are needed to focus on multiple cell lines and building animal model to further confirm and support the findings in our study. And the effect of overexpression of PITX2 affects lncRNA GHET1.

## Methods

### Cell culture and transfection

The normal human colonic epithelial cell line, HCoEpiC, and the human colon cancer cell lines, SW480, Caco-2 and LoVo, were obtained from the American Type Culture Collection. Cells were cultured in 10% RPMI-1640 medium (HyClone; Cytiva) supplemented with 10% FBS (Gibco; Thermo Fisher Scientific, Inc.) at 37 °C in a humidified atmosphere with 5% CO_2_.

To establish PITX2-knockdown and lncRNA GHET1-overexpressing SW480 cells, lentiviral particles containing the corresponding plasmids were purchased from Shanghai GeneChem Co., Ltd. Polybrene (Shanghai GeneChem Co., Ltd.) was used to enhance the transduction efficiency.

### Cell Counting Kit-8 (CCK-8) assay

SW480 cells were digested with trypsin (Beyotime Institute of Biotechnology) into a single-cell suspension and cells were then seeded into four 96-well plates at a density of 2 × 10^3^ cells/well. Following cell adherence, the CCK-8 reagent (Dojindo Molecular Technologies, Inc.) was diluted in culture medium and added into each well. Cells were then incubated at 37 °C for 1 h. The absorbance of each well was measured at 0, 24, 48 and 72 h at a wavelength of 450 nm using a spectrophotometer (Thermo Fisher Scientific, Inc.).

### Colony formation assay

Prior to the colony formation assay, cells were digested with trypsin into a single-cell suspension. Subsequently, cells were resuspended in culture medium, plated into 60-mm culture dishes at a density of 300 cells/dish and cultured at 37 °C in an incubator with 5% CO_2_ for 2 weeks. Following incubation, cells were fixed with 70% ethanol solution and stained with crystal violet (Thermo Fisher Scientific, Inc.). Finally, the number of formed colonies was calculated under an inverted phase contrast microscope (Olympus Corporation, Magnification, ×10).

### Wound healing assay

Cells in a single-cell suspension were seeded into 6-well plates at a density of 5 × 10^5^ cells/well. Subsequently, the monolayer of cells was scratched with a 200-µl sterile pipette tip and the cells were incubated in serum-free DMEM for 24 h at 37 °C. The migratory distance of the cells was observed under a light microscope (magnification, ×100; Olympus Corporation) and analyzed using ImageJ version 1.49 software (National Institutes of Health).

### Transwell assay

For the cell invasion assay, the upper chambers of Transwell plates (BD Biosciences) were precoated with Matrigel (BD Biosciences) at 37 °C for 2 h, then cells (5 × 10^6^ cells/ml) were seeded into the upper chambers in serum-free medium. DMEM supplemented with 2.5% FBS was plated into the lower chambers. Following culture for 24 h at 37 °C, cells on the upper surface of the Transwell membrane were removed with a cotton swab. Cells on the lower surface of the Transwell membrane were washed with PBS and fixed with 4% paraformaldehyde at room temperature for 30 min. Subsequently, the paraformaldehyde was discarded, and cells were stained with 0.1% crystal violet at room temperature for 15 min, prior to being observed using an optical microscope (Olympus Corporation). The total number of cells in each field of view was recorded and the mean number of cells was calculated.

### RT-PCR

Total RNA was collected with the Trizol (Thermo Fisher Scientific, USA) method. Then we used a reverse transcription kit (Takara, Japan) to reverse transcribe these RNAs into cDNA. Next, ABI 7500 system (Thermo Fisher Scientific, USA) was used for the amplification of these cDNA. And 2^−ΔΔCt^ method was used for the analysis of the results. The primers used in this study were lncRNA GHET1 forward primer: 5′-CCCCACAAATGAAGACACT-3′ reverse primer: 5′-TTCCCAACACCCTATAAGAT-3′, PITX2 forward primer: 5′-CGGCAGCGGACTCACTTTA-3′ reverse primer: 5′-GTTGGTCCACACAGCGATTT-3′ GDPDH forward primer: 5′-GGAGCGAGATCCCTCCAAAAT-3′ reverse primer: 5′-GGCTGTTGTCATACTTCTCATGG-3′.

### Western blot analysis

Total proteins were extracted from cells using RIPA buffer (Beyotime Institute of Biotechnology) and the protein concentration in each sample was measured using the BCA assay method. Subsequently, the proteins were separated by 10% SDS-PAGE (Beyotime Institute of Biotechnology) and were then transferred onto PVDF membranes (MilliporeSigma). Following blocking with skimmed milk powder, membranes were incubated with primary antibodies at 4 °C overnight. The primary antibodies used were as follows: anti-cyclin D1 (cat. no. ab16663), anti-c-Myc (cat. no. ab32072), anti-MMP-7 (cat. no. ab207299), anti-PITX2 (cat. no. ab221142) and anti-GAPDH (cat. no. ab8245; all from Abcam). The next day, the membranes were washed with PBS-Tween-20 for three times and were then incubated with the corresponding secondary antibodies for 2 h. Finally, the immunoreactive signals were detected by Pierce Western Blotting Substrate (Thermo Fisher Scientific, Inc.). The relative protein expression was quantified using the ImageJ software (National Institutes of Health).

### Luciferase reporter assay

The luciferase reporter plasmids were obtained from Shanghai GeneChem Co., Ltd. A Dual Luciferase Reporter Assay System (Promega Corporation) and commercially available kits were utilized to detect the fluorescence intensity according to the manufacturer's instructions.

### Chromatin immunoprecipitation (ChIP) assay

SW480 cells were treated with 4% paraformaldehyde, washed with cold PBS and collected in a centrifuge tube. Subsequently, cells were centrifuged for 10 min and were first resuspended in cell lysis buffer and then in nuclear lysis buffer. DNA fragmentation was carried out by ultrasound. Then, 5 μg anti-PITX2 antibody (cat. no. ab192495; Abcam) or 5 μg mouse IgG (cat. no. sc-2025; Santa Cruz Biotechnology, Inc.) were used for immunoprecipitation. The antibodies were mixed with dilution buffer supplemented with magnetic beads (Thermo Fisher Scientific, Inc.). The DNA fragment–antibody complexes were incubated at 4 °C overnight. The next day, the immunoprecipitated complexes were treated with ChIP elution buffer and the eluted DNA was subjected to qPCR.

### Statistical analysis

All experiments were repeated at least three times and the data were analyzed with GraphPad Prism 7 software (GraphPad Software, Inc.). The results are presented as the mean ± SD. The differences between two groups were compared by one‐way ANOVA (analysis of variance) with Tukey–Kramer post hoc test and *p* < 0.05 was considered to indicate a statistically significant difference.

## Data Availability

The datasets used and/or analyzed during the current study are available from the corresponding author on reasonable request.

## Abbreviations

PITX2: Paired-like homeodomain transcription factor 2
lncRNA: Long non-coding RNA
